# The Evolution of Opportunistic Behavior of Participating Subjects during the Operation Period of PPP Projects

**DOI:** 10.1155/2022/8450529

**Published:** 2022-04-14

**Authors:** Dan Peng, Xiaoye Zeng

**Affiliations:** ^1^College of Road and Bridge Engineering, Hunan Communication Polytechnic, Changsha, Hunan 410132, China; ^2^Department of Engineering Management, School of Civil Engineering, Central South University, Changsha, Hunan 410083, China

## Abstract

Most of the public-private partnership (PPP) projects have entered the operational period in China. Due to information asymmetry, opportunistic behavior exists in the operation period of PPP projects. The opportunistic behavior of the participating subjects is an obstacle to the success of PPP projects and one of the root causes of low project performance. To investigate the evolutionary law of this behavior, the payment matrix of the strategic interaction between the investors and the government is constructed based on evolutionary game theory. Using MATLAB to simulate the evolutionary state and through the analysis of the evolutionary behavior of the interaction process, the influence of individual strategy choice on group behavior is revealed. The results show that the path evolution system of opportunistic behavior during the operation period of PPP projects can converge in two states, “good” and “bad,” and the determining factor is the relative returns of investors under various strategies. Reasonable incentives and penalties and reduced regulatory costs can effectively discourage opportunistic behavior. By regulating the parameters, the path evolution of opportunistic behavior can be optimized, and the group behavior can be induced to a good state. The research results can provide a reference for reducing the opportunistic behavior of participating subjects and improving the success rate of the PPP projects.

## 1. Introduction

Providing efficient social public services is one of the important functions of government, and as society continues to develop, public demand for the quantity and quality of public services is growing rapidly [1]. It is predicted that for every increase in urban population, infrastructure investment increases by at least 10,000 CNY [2]. According to the Ministry of Finance of China, the urbanization rate of the country will increase by nearly 5% between 2015 and 2020, thus driving the demand for urban infrastructure investment of over 40 trillion CNY [3]. There is no doubt that the Chinese government has limited revenues and cannot afford such a huge funding requirement. Traditional procurement methods cannot tackle the challenges faced by the government [4]. Compared with the traditional procurement model, the PPP model has the advantages of enriching funding sources, dispersing and reducing government risks, introducing social capital technology and efficient services, and optimizing resource allocation, which has been strongly supported and promoted by the government [5].

PPP refers to a long-term contract between a government and a private party for the provision of public assets or services, in which the private party bears significant risks and management and remuneration is linked to performance [6]. Compared with traditional public utility delivery methods, the PPP model has obvious advantages such as economic savings and improved efficiency, which can solve the problems of insufficient financial resources and inefficient supply of public assets and realize the advantageous integration of the public and private sectors [7]. In view of the obvious advantages of PPP in infrastructure construction and social service provision, it has been widely accepted worldwide in the past 20 years, and more and more countries, from developed to developing countries, have introduced the PPP model [8]. The UK pioneered the concept of PPP and actively privatized public services, mainly through the private finance initiation (PFI) approach [5]. Since then, PPP has been widely adopted in several countries, and its concept and experience have been strongly promoted over the world. Infrastructure PPP projects have grown rapidly over the past two decades, with most countries actively adopting the PPP approach.

The development of the PPP model in China is divided into three stages. In the first stage since the reform and inception in 2003, the exploration of PPP mainly remains at the theoretical level. Start the BOT model for operation and enhance the understanding of PPP in the exploration of BOT [9, 10]. The second stage is from 2004 to 2012. During this period, the PPP model was implemented rapidly, from expanding the preliminary pilot projects to comprehensively promoting the pilot course. The rapid development of PPP during the decade and the incentive policies and measures of relevant departments have gradually improved the regulatory framework of PPP, which in turn improves the promotion and development of related projects [3]. The third stage is the standardization stage of the PPP model. Since 2013, PPP has been vigorously promoted, the development of PPP mode in China has entered a period of explosive growth, and China has become the PPP market with the largest influence and scale in the world. In 2013, there were 80 PPP projects in the first batch of China Development and Reform Commission projects, including many transportation facilities such as highways and ports [11]. At present, most of China's PPP projects have entered the operation stage. The operation period is the core stage of PPP projects and is an important stage to test the success of adopting the PPP model. While the widespread adoption of PPP has been remarkably successful, there are some challenges due to the complexity of the PPP model. The conflict between project stakeholders often leads to project failure in the operation period [12, 13]. The reason for that is that the expectation and benefits of project outcomes vary depending on the stakeholders [14–16].

The goal of government departments is to maximize the public interest of the society, that is, through PPP projects to achieve the demands of public use and improve public benefits [17]. While private investors pursue the maximization of their interests and consider the interests first throughout the project process, considering how to increase revenue and reduce costs to improve the profit return of the project [18]. A typical PPP project includes a series of stages such as bidding, construction, operation, and transfer [19, 20]. In the whole life cycle of PPP projects, the operation phase lasts for a long time, which may last for decades [21, 22]. The government has more resources to dominate partnerships through contracts in the early stages of PPP projects. In particular, the government has an advantage in all aspects of bidding, construction, and initial operation stages [23]. However, during the long operating period, the investors have the responsibility for the operation and maintenance of the project, with relatively little government involvement. Investors are more likely to have an information advantage than government agencies, which makes investors pursue their interests and act opportunistically to damage the project during the operation period, especially in the late operation period [3]. When the operating period ends, the franchising of the project will be transferred back to the government. In the pursuit of maximum profit, investors may overuse facilities without proper maintenance. Alternatively, in an effort to save on operating costs, investors in other types of infrastructure, such as highways, tend to neglect major overhauls and routine road maintenance or pursue actions such as reducing the number of service stations and rest areas [19]. Such opportunistic behaviors can be seriously detrimental to the public interest and the success of a PPP project [24]. Therefore, it becomes urgent to examine methods by which the government can effectively address and prevent such opportunistic behaviors.

In PPP projects, the government is a participant in the project, and as an administration, it also plays the role of regulator. The private investors pursue the project for profit; it does not aim at the public interest, so the government department must supervise the project to maintain the social benefit [25]. The purpose of this study is to analyze the strategic choice of opportunistic behaviors between government and investors based on evolutionary game theory during the project operation period from the perspective of regulators. Through the analysis of the evolutionary behavior of the interaction process, the influence of individual strategy choice on group behavior is revealed.

This study makes full use of the results of the evolutionary game to guide the practice of PPP projects and promote the construction of operation management mechanisms for PPP projects. Meanwhile, it broadens the application field of evolutionary games and complements the method of PPP operation management mechanism research. It is conducive to guiding PPP participating subjects to reduce opportunistic behavior during the operation period and establish a positive management mechanism, which improves the success rate of PPP projects and promotes the feasibility of the application.

## 2. Literature Review

### 2.1. Public-Private Partnerships

The PPP model originated in the United Kingdom. Norment [26] describes the origin of the PPP model, in which a 20-year “Design-Build-Finance-Operate (DBFO)” contract was signed between the government and the private investors in 1998 for the construction of the Manchester building in the UK. At that time, the PPP model was called the “private finance initiative (PFI).” The PPP model has been applied in the fields of highways, hydropower plants, infrastructure, government office buildings, and urban rail transportation [27–31].

The PPP is defined differently by each country or international organization, with the core element being a “partnership” between government and private capital [6, 32, 33]. PPP is an institutional innovation for infrastructure construction. The introduction of private capital in infrastructure construction under the PPP pattern can promote great progress in labor productivity and service quality and promote the competitiveness of infrastructure construction, thus significantly improving the quality of infrastructure services [34, 35]. Infrastructure construction under the PPP pattern solves the inefficient state of traditional public assets supply, and private capital is stronger than the government in operational efficiency and service quality, thus promoting the improvement of public service quality [36–38]. PPP is also a kind of financial system innovation, which provides a good opportunity for social capital to participate in public services. The success or failure of the PPP operation depends on the financing model of project funds, and financial risk management techniques can rationalize the allocation of risks in infrastructure construction. It can be split in time and divided into different segments in space. This facilitates the access of social capital to different steps and mitigation according to their risk appetite, resulting in various forms of leveraged financing, structured financing, and lease financing [39]. As the risk is determined in the contracts of each step, it allows these contracts to circulate in the market, meeting the need for the social capital exit while performing the basic function of risk diversification in the financial market [40].

PPP projects are a set of contractual relationships between the participants, and a large body of literature has studied the operation of PPP projects from a contractual perspective [41–43]. From the contractual nature of PPP projects, the incentive mechanism and compensation problems in PPP projects are analyzed by using principal-agent theory and game theory [44–47]. In PPP projects, the government is a participant in the project; meanwhile, as an administrative agency, the government department acts as a supervisor [48]. The research on the PPP project regulation mainly focuses on the supervision mechanism and supervision behavior. Kumaraswamy and Zhang [49] argued that the success of PPP projects should not rely entirely on the conscious behavior of project investors to ensure the success of PPP projects and pointed out that an independent third-party organization should be established to supervise and manage the construction and operation of PPP projects. Petersen [50] compared and analyzed the supervision mechanisms of PPP projects in two countries, Denmark and Ireland. The study finds that the supervision mechanisms of PPP projects are significantly different under different political and economic systems.

### 2.2. Opportunistic Behavior

Opportunism is a self-interest tendency of humans, which usually assumes that the rational “economic man” is trying to achieve his maximum utility, and this assumption determines the risk of opportunism in various contractual relationships [51]. The manifestations of opportunists can be divided into two types of positive forms, such as aggression and forced concessions, and negative forms, such as refusal to adjust and escape responsibility, and they coexist with each other in long-term economic activity [52]. The specific manifestations are mainly in the form of breach of contract, concealment of misleading and distorted information, refusal to adjust itself to changes in the environment, incomplete performance or evasion of due obligations, and others [53]. Opportunistic behavior has drawn increasing attention from industry and government [54, 55]. There is also a great deal of opportunism in construction projects [56], especially in PPP projects [57]. Lohmann and Rötzel [58] explored the opportunistic behavior of PPP projects during the contract negotiation phase. Liu et al. [59] based on the contractual relationship between the government and private investors in PPP projects, principal-agent models in the presence of opportunistic tendencies in private investors were constructed to analyze the incentive mechanism for inhibiting investors' opportunistic tendencies in PPP projects. Ping Ho et al. [23] proposed an opportunism-focused transaction cost analysis of PPP projects to supplement the current practice of PPP feasibility analysis. Nguyen and Garvin [60] explored the tension between public sector control and concessioner empowerment over the project lifecycle by examining how 23 PPP contracts in the US highway sector were structured.

### 2.3. Evolutionary Game Theory

Game theory was originally proposed by Morgenstern and Neumann [61], and Nash [62] proved the existence of Nash equilibrium, refined the classical game model, and promoted its development. Classical game theory explores conflict as well as cooperation between perfectly rational decision-makers by building mathematical models [63]. Game theory is the most effective decision theory and economic analysis tool for studying cooperation, conflict, and interaction in the decision-making process [64]. Conflict and cooperation are very common in public-private partnerships, and game theory can effectively analyze these issues [65]. Biological evolution was the source of evolutionary game theory [66]. Nash [67] proposed a more complete theory based on evolutionary game theory. Subsequently, Smith and Price [68] introduced an important concept, which is the evolutionary stable strategy (ESS). Another fundamental concept of evolutionary game theory is the replicator dynamics equation [69]. Replicator dynamics equations and ESS are the core concepts of evolutionary games, which lay a solid foundation for the development of evolutionary game theory.

Evolutionary game theory is often used to analyze the decision-making process among participants [70–72]. The strategy selection problem of some PPP projects is can be expressed as a game between the government and private investors [73]. In the analytical framework of classical game theory, the government and investors are regarded as perfectly rational subjects. However, in real life, both parties are considered to be finite rational subjects. The government and investors seldom find the best choice strategy at the beginning, but both parties are constantly learning and adjusting to optimize their initial strategies through repeated trials. Therefore, the behavioral strategies of government departments and investors in the operation period of PPP projects are consistent with the analytical framework of the evolutionary game.

Currently, most scholars focus on the research of PPP project risk management as well as the concession period and concession price. For the PPP operation period, scholars have mostly discussed the issue from the perspective of risk. Few scholars have studied the opportunistic behavior of participating subjects during the operation of PPP projects, and there is a lack of pertinence in the research process. As for the construction of the PPP project operation management mechanism, the existing researches ignore the influence of investors' interests and behavioral choices, and the research on PPP operation management mechanism using evolutionary game theory still has gap.

## 3. Evolutionary Game Model Construction

### 3.1. Model Assumptions

During the 10–30 years of operation of PPP projects, the behavior of regulators and investors can be regarded as a dynamic game. To ensure the scientific and objective nature of this study, the following assumptions are made.

**Hypothesis 1 .**

The groups involved in the game are two types of independent groups: government and investors. The strategy space of individuals in both types of groups is the common knowledge of all individuals. The group members make different choices based on the value assessment of different strategy results and make strategy adjustments dynamically based on the actual results of different strategies.

**Hypothesis 2 .**

There are two behavioral strategies available to investors in the operation phase of PPP projects: one is not to act opportunistically, which means to operate the project normally in accordance with the relevant contracts and policies, and regulations. Another option is to adopt opportunistic behaviors, such as not performing program maintenance, updating equipment, and reducing service quality. The set of alternative behavioral strategies for investors are assumed {to act opportunistically, not to act opportunistically}. Government departments also have two strategies: one is to seriously supervise the behavior of investors in the operation phase of PPP projects, and the other is not to supervise the behavior of investors to save supervision costs. Hence, the behavioral strategies of government are assumed {to supervise, not to supervise}.

**Hypothesis 3 .**

Government departments have a 100% success rate in detecting opportunistic behavior of PPP project investors during the operation period through supervision. Government supervision will incur a corresponding cost, and government departments will penalize investors for opportunistic behavior in the operation period of the project.

**Hypothesis 4 .**

In the game process, the impact of the choice of behavioral strategies of both parties on their respective gains is considered. Both sides of the game also choose their behavioral strategies based on the benefits they bring, without considering the changes in benefits brought about by other external factors of the environment.

**Hypothesis 5 .**

For the sake of analytical simplicity, it is assumed that the combinators are all profit-oriented and must wish to pursue profit maximization, not always complying with laws and regulations. The government is strictly involved in regulation and incentives according to the system, with certain incentives for investors to operate and produce strictly according to the contract. Once opportunistic behavior is detected, the incentives will be canceled and penalized.

### 3.2. Parameter Setting and Payoff Matrix Building

Based on the above assumptions and analysis, the payoff matrix can be created for the game process of government supervising the opportunistic behaviors of investors, as shown in Table 1.

The relevant parameters (non-negative) of the payoff matrix in Table 1 are explained as follows:

 
*α* – incentive coefficient for investors who do not act opportunistically
 
*β* – benefit coefficient for investors who act opportunistically
 
*L* – normal production income of investors during the operation period of PPP projects
 
*C* – supervision cost of government
 
*X* – the benefit of government when the investors do not act opportunistically
 
*D* – losses caused by lack of government supervision
 
*F* – punishment imposed by the government through supervising for opportunistic behavior of investors, such as fines

During the operating period of a PPP project, the conditions that incentivize investors to choose opportunistic behavior are when *β* > *α*. In other words, investors have the impulse to act opportunistically only if the benefits of doing so are greater than the benefits of not doing so. Meanwhile, when *αL* > *βL* − *F*, after the government penalty, the net benefit to the investors from choosing opportunistically is smaller than the net benefit from not choosing opportunistically. Otherwise, government supervision is meaningless. At the same time, only when *X* > *D* and *X*> *C*, that is, the cost incurred by the government in supervising and the loss caused by the lack of supervision are less than the benefit of government when the investors do not act opportunistically. Otherwise, the government will lose the incentive to supervise.

### 3.3. Modeling

According to the payoff matrix of the game between the government and the investors above and combined with the theory and methods of evolutionary game analysis, the gains of both sides of the game can be analyzed. Assuming that at the initial time of the PPP project operational period, the proportion of the investor group that does not act opportunistically is *p*, and the proportion that acts opportunistically is (1 − *p*). The percentage of government choose supervision is *q*, and then the percentage of government choosing not to supervision is (1 − *q*).

The expected payoff of the investors choosing the strategy of not to act opportunistically is *U*_1_, and the expected payoff of the investors choosing the strategy of act opportunistically is *U*_2_. The mean payoff of the investor group is $\bar{U}$. The representative equations are as follows:

(1) $$\begin{array}{c} U_{1}=\alpha L, \end{array}$$

(2) $$\begin{array}{c} U_{2}=\beta L-qF, \end{array}$$

(3) $$\begin{array}{c} \bar{U}=pU_{1}+\left(1-p\right)U_{2}. \end{array}$$

Similarly, the expected payoff of government choosing the strategy of who to supervise is *V*_1_, and the expected payoff of government choosing the strategy of who not to supervise is *V*_2_. The mean payoffs of the group of government are $\bar{V}$. The representative equations are as follows:

(4) $$\begin{array}{c} V_{1}=p\left(-C+X\right)+\left(1-p\right)\left(-C+F\right), \end{array}$$

(5) $$\begin{array}{c} V_{2}=q\left(X-D\right), \end{array}$$

(6) $$\begin{array}{c} \bar{V}=qV_{1}+\left(1-q\right)V_{2}. \end{array}$$

Based on the Malthusian dynamic equations, according to equations (1)–(6), the replication dynamic equations for the strategic interactions of the investors and the government, respectively, are constructed as follows:

(7) $$\begin{array}{c} \left\{\begin{array}{c} F\left(p\right)=\frac{\text{d}p}{\text{d}t}=p\left(U_{1}-\bar{U}\right)=p\left(1-p\right)\left(\alpha L-\beta L+qF\right),\\ F\left(q\right)=\frac{\text{d}q}{\text{d}t}=q\left(V_{1}-\bar{V}\right)=q\left(1-q\right)\left[-C+F+p\left(D-F\right)\right], \end{array}\right. \end{array}$$

where *t* is the time and d*p*/d*t* and d*q*/d*t*, respectively, are the rates of change of the proportion of the investors and government, which do not act opportunistically and supervise. Equation (7) allows us to study the evolution of the strategic engagement behavior of investors and regulators. On the basis of the method proposed by Friedman [74], the stabilization conditions at the equilibrium point of the system can be derived using the Jacobi matrix of the system. The Jacobi matrix *J* of equation (7) is denoted as follows:

(8) $$\begin{array}{c} J=\left[\begin{array}{cc} \frac{\text{d}F\left(p\right)}{\text{d}p} & \frac{\text{d}F\left(p\right)}{\text{d}p}\\ \frac{\text{d}F\left(q\right)}{\text{d}p} & \frac{\text{d}F\left(q\right)}{\text{d}p} \end{array}\right]=\left[\begin{array}{cc} \left(1-2p\right)\left(\alpha L-\beta L+qF\right) & p\left(1-p\right)F\\ q\left(1-q\right)\left(D-F\right) & \left(1-2p\right)\left[-C+F+p\left(D-F\right)\right] \end{array}\right]. \end{array}$$

The row equation is written as Det*J*, and the trace is written as Tr, which are shown as the following equations:

(9) $$\begin{array}{c} \text{Det}J=\left(1-2p\right)\left(1-2q\right)\left(\alpha L-\beta L+qF\right)\left[-C+F+p\left(D-F\right)\right]-pqF\left(1-p\right)\left(1-q\right)\left(D-F\right), \end{array}$$

(10) $$\begin{array}{c} \text{Tr}=\left(1-2p\right)\left(\alpha L-\beta L+qF\right)+\left(1-2q\right)\left[-C+F+p\left(D-F\right)\right]. \end{array}$$

## 4. Evolutionary Game Model Analysis

### 4.1. Equilibrium Points and Their Stability Analysis

On the plane *M*={(*p*, *q*)*|*0 ≤ *p*, *q* ≤ 1}, five possible equilibrium points (0,0), (0,1), (1,0), (1,1), and (*p*^*∗*^=*D* − *F*/*X* − *F*, *q*^*∗*^=*βL* − *αL*/*F*) of the evolutionary game can be obtained. Its local stability analysis was obtained, and the results are shown in Table 2.

**Figure 1:**
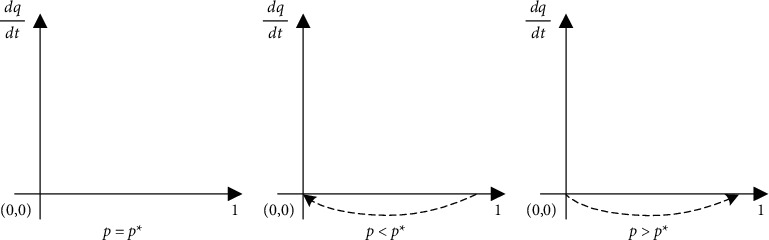
Diagram of the dynamic evolution of opportunistically acted by the investors.

**Figure 2:**
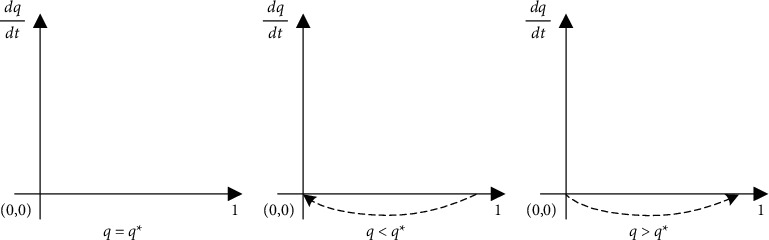
Diagram of the dynamic evolution of supervision acted by the government.

From Table 2 with the constraints *S*(*C* − *F*/*D* − *F*, *βL* − *αL*/*F*) as saddle points, the analysis shows that among the five local equilibrium points, only (0, 0) and (1, 1) have local equilibrium and are ESS, corresponding to the strategies of {not to act opportunistically, to supervise} and {act opportunistically, not to supervise}. In addition, there are two unstable equilibria and one saddle point. The diagram of this evolutionary model depicts the dynamic evolution of the game between the two parties, as shown in Figures 1 and 2.

**Figure 3:**
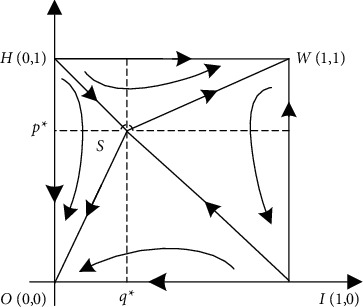
Diagram of the dynamic evolution of the equilibrium points.

Placing the dynamic evolution diagrams of Figures 1 and 2 in a plane coordinate yields Figure 3. The fold line connecting the two imbalance points H and I and the saddle point, and S in the diagram can be seen as the critical line where the system converges to different modes. When the initial state is in the HSIO region, the system all converges to the {act opportunistically, not to supervise} mode; the investor chooses to act opportunistically; and the regulator does not regulate well, which is a bad lock-in state. When the initial state is within the HSIW region, the system converges to the mode {not to act opportunistically, to supervise}, the investor chooses not to act opportunistically, and the owner is well regulated, which is a good state.

The above analysis shows that the outcome of the game between the investor and the regulator may eventually stabilize at {act opportunistically, not to supervise} or at {not to act opportunistically, to supervise}. Which path or direction the dynamic game will evolve along is closely related to the changes in the initial values of the parameters that make up the payoff matrix for both sides of the game.

### 4.2. Numerical Experiments and Results Analysis

Based on the constraints and replicated dynamic equations, MATLAB software is applied to experimentally analyze the behavioral evolution process of PPP projects during the operation period. The impact of the changes in parameters such as initial population proportion, incentive coefficient, benefit coefficient, production income, penalty, and benefit of government for choosing a certain strategy on the evolutionary results is analyzed. The reference values are *α*=0.6, *β*=0.8, *L*=10, *F*=4, *C*=6, *D*=8, and *X*=9.

#### 4.2.1. The Effect of Initial Population Change for Choosing a Certain Strategy on the Evolutionary Outcome

**Figure 4:**
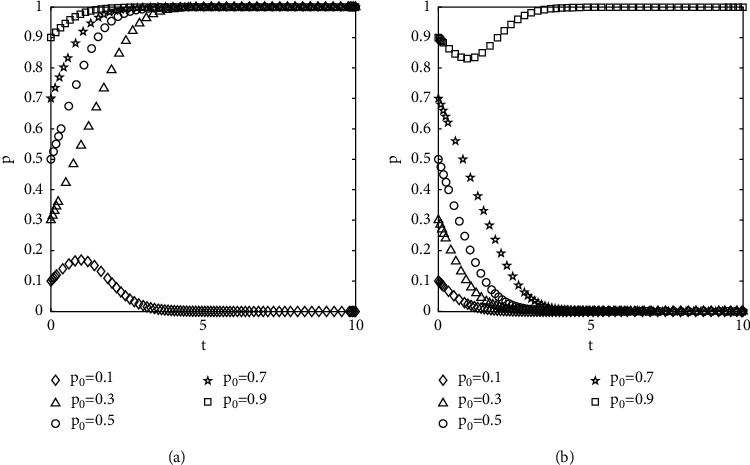
Diagram of the effect of different initial proportions of groups choosing different strategies on evolutionary results: (a) *q*_0_ = 0.8 and (b) *q*_0_ = 0.2.

The numerical results are shown in Figure 4, where *p*_0_ and *q*_0_ denote the initial proportions of the populations of the investors selected not to opportunistically and the government-selected to supervise, respectively. It can be seen from Figure 4 that the curves of different initial conditions do not intersect and overlap until they reach the convergence state. The speed of convergence is affected by the initial proportion of population selection, and the closer to the equilibrium state, the faster the speed of convergence. The evolution of the behavior of investors is affected by the initial proportion of selection and the strength of regulation by the government.

#### 4.2.2. The Effect of the Change in the Incentive Coefficient on the Evolutionary Results

**Figure 5:**
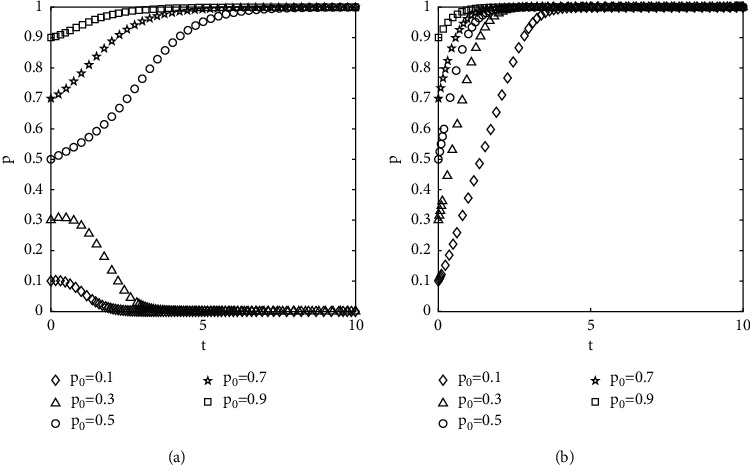
Diagram of the effect of variation of incentives coefficient on evolutionary results: (a) alpha = 0.5 and (b) alpha = 0.7.

The proportions of the government-selected to supervise are set at 0.8, and all parameters except the incentive coefficient are kept constant. The results of the numerical experiments are shown in Figure 5. Comparing Figures 5(a)with 5(b), it can be seen that the system evolves to a good state with the increase of the incentive coefficient. It shows that the full affirmation of the government and the financial incentives within the cost allowed can actively motivate the investors and increase the possibility of the system evolving to a good state.

#### 4.2.3. The Effect of Change in the Coefficient of Interest on Evolutionary Results

**Figure 6:**
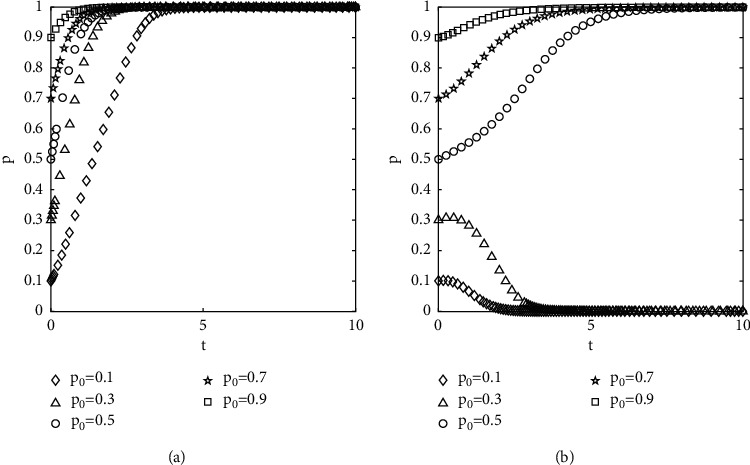
Diagram of the effect of the change in the coefficient of interest on the evolutionary results: (a) beta = 0.7 and (b) beta = 0.9.

All parameters except the benefit coefficient remain unchanged, and proportions of the government selected to supervise are set at 0.8. The benefit of opportunistic behavior is the largest driver of that behavior. The results of the numerical experiment are shown in Figure 6. If the benefits are small, the system evolves to the mode of {not to act opportunistically, to supervise}. As the benefits increase, the driving force increases and changes in the direction of evolution of groups that do not act an initially small proportion opportunistically. As the interest increases, the driving force increases, and the evolutionary direction of the group that does not adopt opportunistic behavior is changed to a state of “opportunistic behavior without careful supervision.” However, for groups with a large initial proportion of non-opportunistically, the convergence rate is slowed down even if the interest drive is significant. However, under the constraint of group norms, the group still evolves to the direction of {not to act opportunistically, to supervise}.

#### 4.2.4. The Effect of the Change in the Penalty on Evolutionary Results

**Figure 7:**
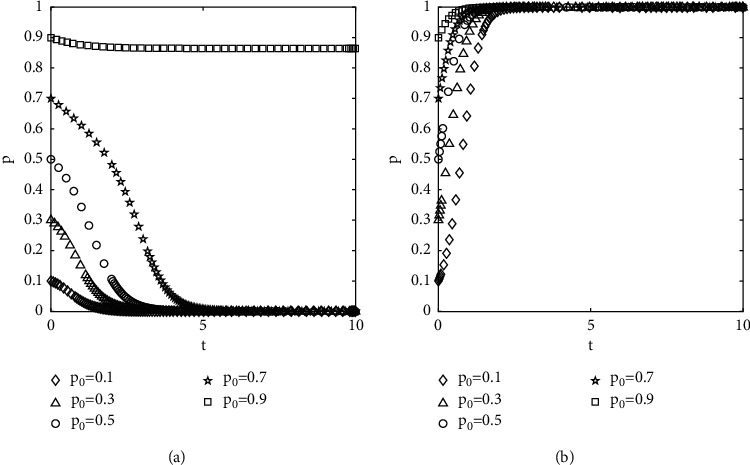
Diagram of the effect of the change in the penalty on the evolutionary results: (a) *F* = 2 and (b) *F* = 6.

All parameters except the penalty are kept constant, and proportions of the government selected to supervise are set at 0.8. The results of the numerical experiment are shown in Figure 7. When the penalty is small, the group, driven by collusive interests, makes the system evolve in the direction of {act opportunistically, not to supervise}. When the penalty is increased, the group evolves in the direction of {not to act opportunistically, to supervise}, which means that increasing the penalty is a powerful measure to restrain the group's behavior.

#### 4.2.5. The Effect of Change in Supervision Costs on the Evolutionary Results

**Figure 8:**
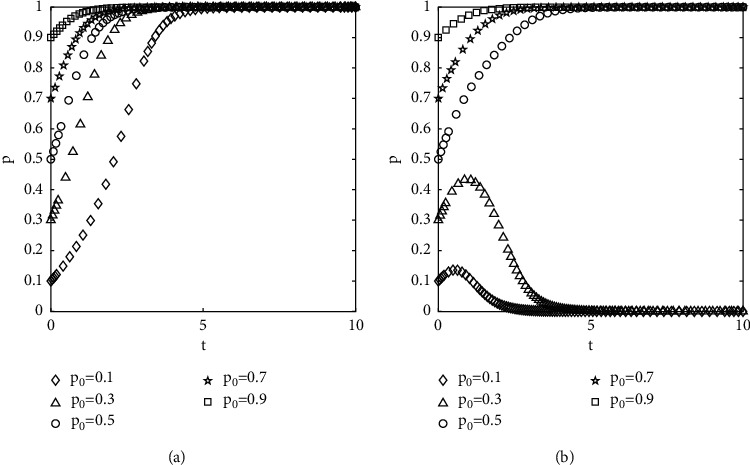
Diagram of the effect of the change in the supervision cost on the evolutionary results: (a) *C* = 5 and (b) *C* = 7.

All parameters except the cost of supervision are kept constant, and the proportions of the government-selected to supervise are set at 0.8. The results of numerical experiments are shown in Figure 8. When the cost of supervision is low, the system evolves in the direction of {not to act opportunistically, to supervise}. Conversely, the increase in supervision costs will reduce the incentive of the government to work. The evolutionary curve of the group with a low percentage of the initial choice of not acting opportunistically rises and then falls, converging to a bad state. It shows that when the initial proportion of groups choosing the “not to act opportunistically” strategy is not dominant, the effect of the government's supervision is not significant. Although it has a short time effect and evolves to a good state. However, as time passes, the segment evolves to a bad state under the effect of gaming psychology

#### 4.2.6. The Effect of Loss Change due to Lack Of Government Supervision on the Evolutionary Results

**Figure 9:**
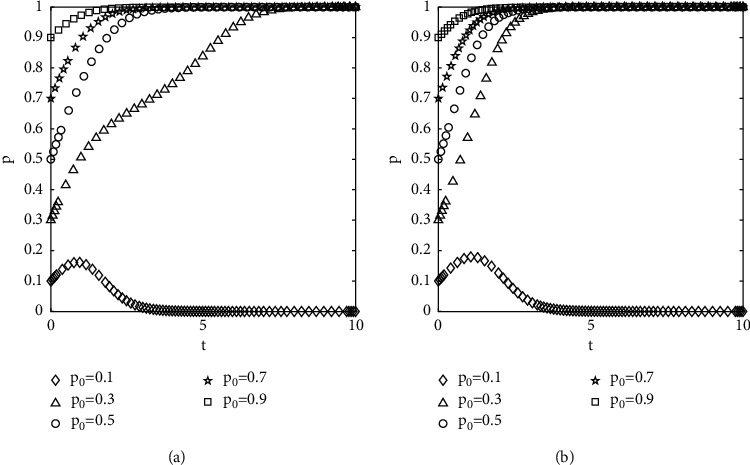
Diagram of the effect of loss changes on evolutionary results for the lack of government's supervision: (a) *D* = 7 and (b) *D* = 9.

Except for the loss due to lack of government supervision, other parameters remain unchanged, and the proportions of the government selected to supervise are set at 0.8. The results of the numerical experiment are shown in Figure 9. It can be seen that the change of the loss of the government has little effect on the evolution of the system, but the loss increases, the faster the system evolves to a good state.

## 5. Discussion

The results of the above study show that whether the PPP investors will maintain and operate the project normally during the operation phase, that is, whether they choose not to act opportunistically is positively related to the benefits of maintaining and operating the project normally and the government's penalty for the investors' opportunistically. Whether an investor chooses not to act opportunistically is negatively correlated with the difference between the net benefit received when the investor act opportunistically and the net benefit received when the investor does not act opportunistically. Whether the government supervises the opportunistic behavior of investors in PPP projects is related to the cost of supervision, the penalty when opportunistic behavior is detected and the losses caused by the lack of supervision. The increase in regulatory costs can lead the government to choose not to supervise, and increasing the penalties for opportunistic behavior of investors can help the government strengthen its supervision efforts.

When the cost of supervision is high for the government, then the government tends to choose not to supervise. Investors tend to choose to act opportunistically when the extra benefits they get from acting opportunistically are higher than the penalties they receive. In this way, the government and investors form a cyclical behavior pattern during the operation period of PPP projects. This cyclical phenomenon explains, to some extent, why opportunistic behavior is repeated emergence in the operation of real projects. The following insights are provided in response to the above analysis.

### 5.1. Establish a Reasonable Incentive and Restraint System

Appropriately increase the penalties for opportunistic behavior of investors in projects during the operational period. Establish a comprehensive PPP project database to give an evaluation of investors' cooperation in the projects. Focus on the record of their opportunistic behaviors so that investors who have taken opportunistic behaviors lose the opportunity to participate in other subsequent PPP projects. In addition, the government should also establish an incentive-compatible payment mechanism to encourage investors' active efforts, closely linking project payment mechanisms with performance evaluation, for instance, paying according to output and performance.

### 5.2. Reduce the Cost of Government Supervision

Reduce the cost of government supervision over the opportunistic behavior of investors during the operation period. The government can set up a special PPP project supervisory institution, which is responsible for supervising the whole lifespan of the project from project establishment to transfer. To solve the current situation of unclear supervision subjects and wasteful overlapping of supervision resources at this stage.

### 5.3. Create a Scientific Supervision System

The government should construct a social supervision guarantee system and improve public participation in the supervision mechanism, including improving the incentive system to encourage and support the public to supervise the behavior of investors. Promote public participation in supervision and reduce public supervision costs through the website, dedicated telephone lines, and other means. By constructing a communication platform for PPP projects and establishing an open and transparent project information release channel, it will facilitate social supervision and motivate enterprises to control costs and improve service levels to protect the interests of the government and the public.

## 6. Conclusion

By constructing an evolutionary game model of opportunistic behavior of investors and regulators during the operation period of PPP projects and analyzing the characteristics of their evolutionary process and stable equilibrium state, we get that the system converges into two states. One is the ideal state, that is, the investor does not take opportunistic behavior and the regulator supervises it seriously. The other is the “bad lock” state, that is, the investor takes opportunistic behavior and the regulator does not supervise seriously. The evolution of the two states is path-dependent, with the initial state and parameter conditions falling into one state and the behavioral participants in the other state disappearing in the evolution.

Through numerical analysis and simulation, it is proposed that to escape from the state of “bad locking,” strengthening the professional ethics and responsibility awareness of staff, formulating assessment mechanisms and reward and punishment measures, and reducing the cost of supervision and assessment are effective solutions, which can better reduce the probability of opportunistic behaviors of participating parties during the operation period of highway PPP projects.

In this study, an evolutionary game is used to study the interaction process between the government and investors during the PPP operation period, and the behavioral evolution strategies of both parties are explored. The simulation analysis reveals the evolution laws of the behaviors. Optimization and improvement strategies are proposed for the evolution results, providing theories and practices for the government to improve the operation and management mechanism.

The project operation process involves three major interests: the government, investors, and the social public. The study only analyzes from the perspective of the government and investors, which makes it impossible to clarify the role played by the social public in the operation of PPP projects. In future research, an evolutionary game model of the government, investors, and the public will be constructed to analyze the behavioral strategy choice and mutual influence of the three parties during the operation period of PPP projects.

## Figures and Tables

**Table 1:** Payment matrix between investors and government.

Investors	Government	
	To supervise	Not to supervise
Not to act opportunistically	*αL*, −*C*+*X*	*αL*, −*D*+*X*
Act opportunistically	*βL* − *F*, −*C*+*F*	*βL*, 0

**Table 2:** Local stability analysis results.

Equilibrium point	Det*J*		Tr		Result
*p*=0, *q*=0	(*αL* − *βL*)(−*C*+*F*)	+	(*αL* − *βL*)+(−*C*+*F*)	−	ESS
*p*=0, *q*=1	(*αL* − *βL*)(*C* − *F*)	+	*αL* − *βL*+*C*	+	Instability
*p*=1, *q*=0	(*αL* − *βL*)(*C* − *D*)	+	−(*αL* − *βL*)+(*D* − *C*)	+	Instability
*p*=1, *q*=1	(*αL* − *βL*+*F*)(*D* − *C*)	+	−(*αL* − *βL*+*F*) − (*D* − *C*)	−	ESS
*p*=*p*^*∗*^, *q*=*q*^*∗*^	(*F* − *C*)(*βL* − *αL*)(*D* − *C*)(*F* − *βL*+*αL*)/*F*(*D* − *F*)	−	0		Saddle point

## Data Availability

All data sets generated for this study have been included in the article.
